# Rotational Power: A New Accelerometer-Derived Metric to Assess Functional Impairment in Multiple Sclerosis

**DOI:** 10.1109/TNSRE.2025.3594540

**Published:** 2025

**Authors:** Guido Mascia, Brett M. Meyer, Josh Cherian, Dheeraj D. Kairamkonda, Jason Fanning, Paige E. Rice, Jacob J. Sosnoff, Andrew J. Solomon, Ellen W. McGinnis, Ryan S. McGinnis

**Affiliations:** Center of Remote Health Monitoring, Wake Forest School of Medicine, Winston-Salem, NC 27101 USA; Medidata Solutions, New York, NY 10014 USA; Center of Remote Health Monitoring, Wake Forest School of Medicine, Winston-Salem, NC 27101 USA; Center of Remote Health Monitoring, Wake Forest School of Medicine, Winston-Salem, NC 27101 USA; Department of Health and Exercise Science, Wake Forest University, Winston-Salem, NC 27109 USA; Department of Health and Exercise Science, Wake Forest University, Winston-Salem, NC 27109 USA; Department of Physical Therapy and Rehabilitation Science, School of Health Professions, University of Kansas Medical Center, Kansas City, KS 66160 USA; Department of Neurological Sciences, College of Medicine, University of Vermont, Burlington, VT 05405 USA; Center of Remote Health Monitoring, Wake Forest School of Medicine, Winston-Salem, NC 27101 USA; Center of Remote Health Monitoring, Wake Forest School of Medicine, Winston-Salem, NC 27101 USA

**Keywords:** Sit to stand, wearables, lower limbs, functional status, fatigue

## Abstract

Multiple sclerosis (MS) is a neurodegenerative disease that affects sensorimotor function, particularly in the lower limbs, leading to increased fatigue and impaired mobility. The 30-second chair stand test (30CST) is a functional assessment that captures muscular strength and endurance in people with MS (PwMS). This study introduces Rotational Power (RP), which is a new metric derived from a single thigh-worn accelerometer, that can serve as a surrogate for body-mass normalized mechanical power during sit-to-stand (SI-ST) and stand-to-sit (ST-SI) transitions. We validate the metric both analytically and clinically in seventeen PwMS who performed a 30CST while instrumented with an accelerometer and a motion-capture system for reference. Analytical validation demonstrated a strong correlation with peak mechanical power for both SIST (r = 0.79) and ST-SI (r = 0.60). Clinical validation revealed strong-to-moderate significant associations between the 95^th^ percentile of peak RP computed across the 30CST and established clinical measures, including the number of repetitions, activity specific balance confidence scale, and modified fatigue impact scale. The same analysis performed on the motion-capture mechanical power showed similar results and concordance in correlation direction. Analysis of acute fatigue during the 30CST showed a significant decline in RP during the task, particularly for concentric transitions. Findings suggest that RP is a valid and practical metric for monitoring functional performance and acute fatigue in PwMS using a single wearable sensor, calling for its future use in free-living settings.

## I. Introduction

Multiple sclerosis (MS) is a neuroinflammatory disease that affects about 3 million people worldwide [1], and whose etiology is still unclear [2]. Persons with MS (PwMS) suffer from an inflammatory cascade leading to demyelination of the axonal processes within the central nervous system, that progressively damages the neurons’ ability to effectively communicate [3], negatively impacting sensorimotor function [2], [3], and increasing the probability of experiencing chronic fatigue [4], [5], [6]. Several studies have demonstrated that the muscle groups in the lower extremities are disproportionately affected by MS [7], [8], with a consequent decline in motor functions involving gait, balance, and functional capacity [7], [9], [10]. Moreover, MS is characterized by a relapsing-remitting nature and significant symptom fluctuations even outside of relapse events [2]. These symptom fluctuations suggest that long-term monitoring of the functional status of PwMS is crucial, as symptoms cannot be reliably assessed at infrequent clinical visits alone.

Among the many tasks necessary to maintain daily independence, the ability to make a postural transition from sitting to standing is of particular interest. In fact, a compromised ability to perform such a postural transition can be prodromic of a progressive decline in functional ability and increase risk of falling [11], [12]. PwMS in general are less active when compared to those without MS, prone to experience chronic fatigue [4], [5], [6], and perform fewer sit-to-stand transitions per day [12], [13], with an upper bound of daily sit-to-stand estimated at 50 for PwMS [12], compared to 71 for adults without balance or mobility impairment [14].

The functional status of PwMS is typically characterized during infrequent clinical visits using patient reported measures of dysfunction and sometimes objective functional assessments [15], [16], [17], [18]. However, symptoms in PwMS are known to fluctuate considerably throughout the day, and from one day to the next. When paired with issues in reporting and recall bias, it is likely that these infrequent assessments are not providing a comprehensive picture of the progression of MS symptoms, and particularly not in such a way as to enable preventative intervention. Moreover, this paradigm can be burdensome for PwMS, and particularly for those living in underserved communities that need to travel long distances to receive specialist neurological services [19]. Objective functional assessments that can be performed and assessed outside of a traditional clinical visit, hold promise for addressing many of these challenges.

The 30-second chair test (30CST) [20] has emerged as a suitable approach for characterizing muscular strength, fatigue, and functional capacity, while reducing the burden and risk for the participant when compared to more demanding alternatives, such as 6-minute walk test [21], [22]. The test has been associated with lower limb strength in young [23] and older adults [20], [24], and provides insights into the fall risk status of PwMS [12], [25]. One output that can be assessed during an instrumented 30CST and that is closely related to strength is mechanical power, which provides a proxy for determining one’s functional ability [8], [26], [27]. Decreased lower limb power generated during sit-to-stand tests has been associated with frailty, poor quality of life, increased risk of falling, hospitalization, mortality, and fatigue in older adults [28], [29], [30], [31], [32]. However, quantifying mechanical power typically requires cumbersome and costly equipment, such as optical motion-capture systems and force platforms, rendering its computation impractical outside of specialized laboratory environments.

Wearable inertial sensors have already been employed for assessing transition characteristics in PwMS in the 30CST [12], [25], [33], and could also be used for quantifying mechanical power. However, very few studies have explored the use of wearable sensors for quantifying sit-to-stand mechanical power [34], [35], [36]. These studies have all relied on inertial measurement units, which contain both an accelerometer and angular rate gyroscope, and have not explored transition power in PwMS. Gyroscopes, unlike accelerometers, are susceptible to time-varying measurement bias [37] and have high power consumption [38], [39], which has traditionally prevented their use for long-term continuous monitoring. As a result, many remote activity monitoring technologies are accelerometer based. To the best of our knowledge, there are not yet validated algorithms for quantifying sit-to-stand mechanical power from just accelerometer data. Similarly, many large studies have included thigh-worn, accelerometer-based activity monitors (e.g., [40], [41], [42]), but methods for extracting sit-to-stand mechanical power from data collected at the thigh have not yet been developed.

Given these needs, in this work we introduce and validate a new quantity for characterizing sit-stand performance, *rotational power* (RP), which can be computed from a single thigh-worn accelerometer. We hypothesize that peak RP will be strongly associated with gold-standard body-mass normalized peak vertical mechanical power as measured by motion-capture during postural transitions. We further hypothesize that RP during the 30CST will capture constructs of balance confidence and fatigue in PwMS because of known relationships between the ability to generate power and daily functioning. Finally, we explore the ability of RP to quantify the acute fatigue effects of the 30CST.

## II. Materials and Methods

### A. Sample Characteristics

This is a secondary analysis [43] that leveraged data from 17 PwMS (13F, 4M; mean ± standard deviation age = 50.5 ± 10.2 years; patient-determined disease steps (PDDS) = 0.9 ± 1.1; disability step: normal = 8; mild = 5, moderate = 2; gait = 2 [44]) with no other cause of balance and/or mobility impairment other than MS. Participants were included only if they were capable of walking unaided, not pregnant and/or breastfeeding, and if they did not present any form of hypersensitivity to adhesives. Sample demographics match the prevalence of MS in the broader population, with rates 3 times higher in females than in males [45]. This study was approved by the UVM IRB (Application Nos. STUDY00000401) and CHRSM (18–0285). After signing the informed consent, participants were asked to complete the activity-specific balance confidence scale (ABC: mean percentage ± standard deviation score = 81 ± 19) [46] and modified fatigue impact scale (MFIS: mean ± standard deviation score = 31 ± 16) [47]. The ABC indicates the perceived confidence in performing common daily activities without losing balance and has been shown to provide a reliable measure of functional ability in PwMS [48]. The MFIS is used to assess the impact of fatigue on daily functioning and includes three sub-scores that further characterize the physical, cognitive, and psychosocial impacts of fatigue [47].

### B. Experimental Setup

**Fig. 1.**
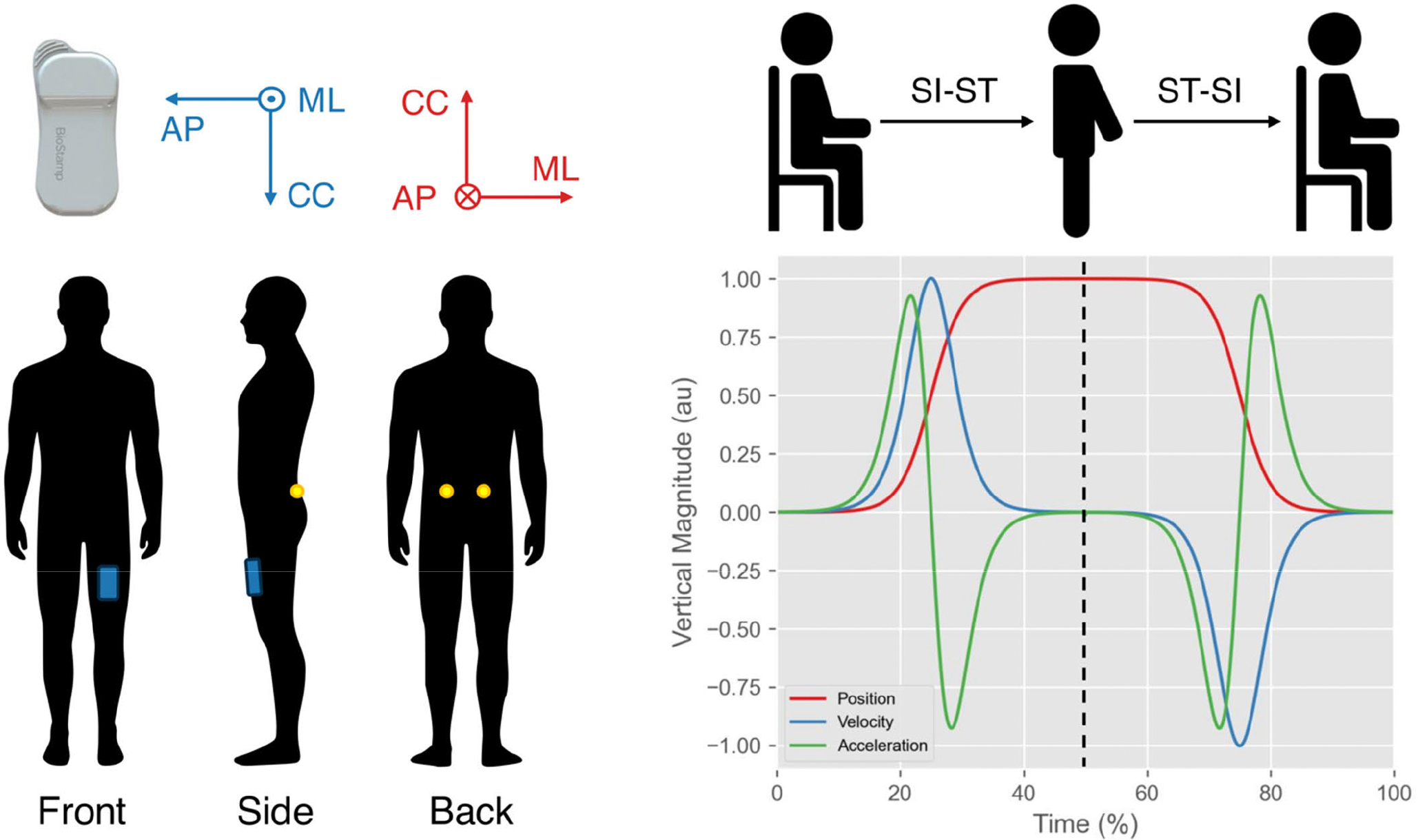
The employed experimental setup (left panel): the Biostamp nPoint sensor with the used right-handed coordinate system employed for the accelerometer (blue) and motion-capture (red) (AP = anterior-posterior; CC = cranial-caudal; ML: medial-lateral); the blue patch represents the accelerometer, placed on the medial portion of the anterior aspect of the left thigh; the yellow circles represent the retro-reflective markers placed on the right and left superior iliac spinae. Synthetic SI-ST and ST-SI cycle (right panel): the red, blue, and green lines represent the vertical displacement, velocity, and acceleration of the body center of mass during a SI-ST (Time < 50%) and ST-SI (Time > 50%). All the magnitudes are expressed as arbitrary units.

Participants completed a variety of in-lab functional assessments as part of a broader study [43] after being instrumented with triaxial wearable accelerometers (Biostamp nPoint, MC10 Inc., Lexington, MA, United States of America; sampling frequency: 250 Hz; range ± 16 g) and a set of retro-reflective markers, whose trajectories were recorded by a motion-capture system (Vicon, Oxford, United Kingdom; sampling frequency: 100 Hz). The battery of tests included timed-up-and-go, 30CST, stair ascent/descent, force plate walking, simulation of activities of daily living, standing balance, and 6-minutes walking test, with no prescribed rest between tasks except for the latter. For the purpose of this study, we focus on the 30CST by considering: i) the accelerometer placed on the medial portion of the anterior aspect of the participant’s left thigh; ii) two retro-reflective markers placed on left and right posterior superior iliac spinae (Fig. 1), whose trajectories were used as an approximation of the participant’s vertical center of mass (CoM) displacement.

After being instrumented, each participant was asked to perform an orthostatic calibration trial, followed by a maximal effort 30CST. The 30CST was executed using a standard chair (height = 43.2 cm = 17 in), asking the participant to maintain their arms crossed on their chest while performing as many sit-to-stand transitions (SI-ST) as possible. The task started as the research assistant gave the “go” signal and ended after 30 seconds. The number of valid repetitions went from the first occurring SI-ST after the “go” signal to the last available completed ST-SI prior to the end of the 30 second period.

### C. Data Processing

The motion-capture trajectories were oversampled to match the accelerometer sampling frequency, so that the two devices could be time-synchronized. The time-lag between the two systems was computed leveraging cross-correlation, with the marker trajectories leveraged to segment each transition, each of which was eventually analyzed individually at their native sampling frequency.

For the motion-capture data, the average vertical displacement (*z)* between left and right posterior superior iliac spinae was computed and low-pass filtered (4^th^-order Bessel Filter, cut-off = 2 Hz). Filter cut-off frequencies were chosen so that the filtered signal retained at least 95% of the original signal energy. Positive and negative peaks were identified, representing the standing (maximum height) and sitting (minimum height) position of the participant, respectively. Each transition was labeled as SI-ST or ST-SI based on the intrinsic characteristics of peak-to-peak vertical displacement, (i.e., increase in height corresponding to a SI-ST). The vertical displacement *z* of the average marker was numerically differentiated to obtain the vertical velocity (*v)* and acceleration (*a)*. Subsequently, the body-mass normalized vertical power (*p)* was computed as the product of *a* and *v*. For each transition, the positive vertical power peak (*VPP*) was computed.

**Fig. 2.**
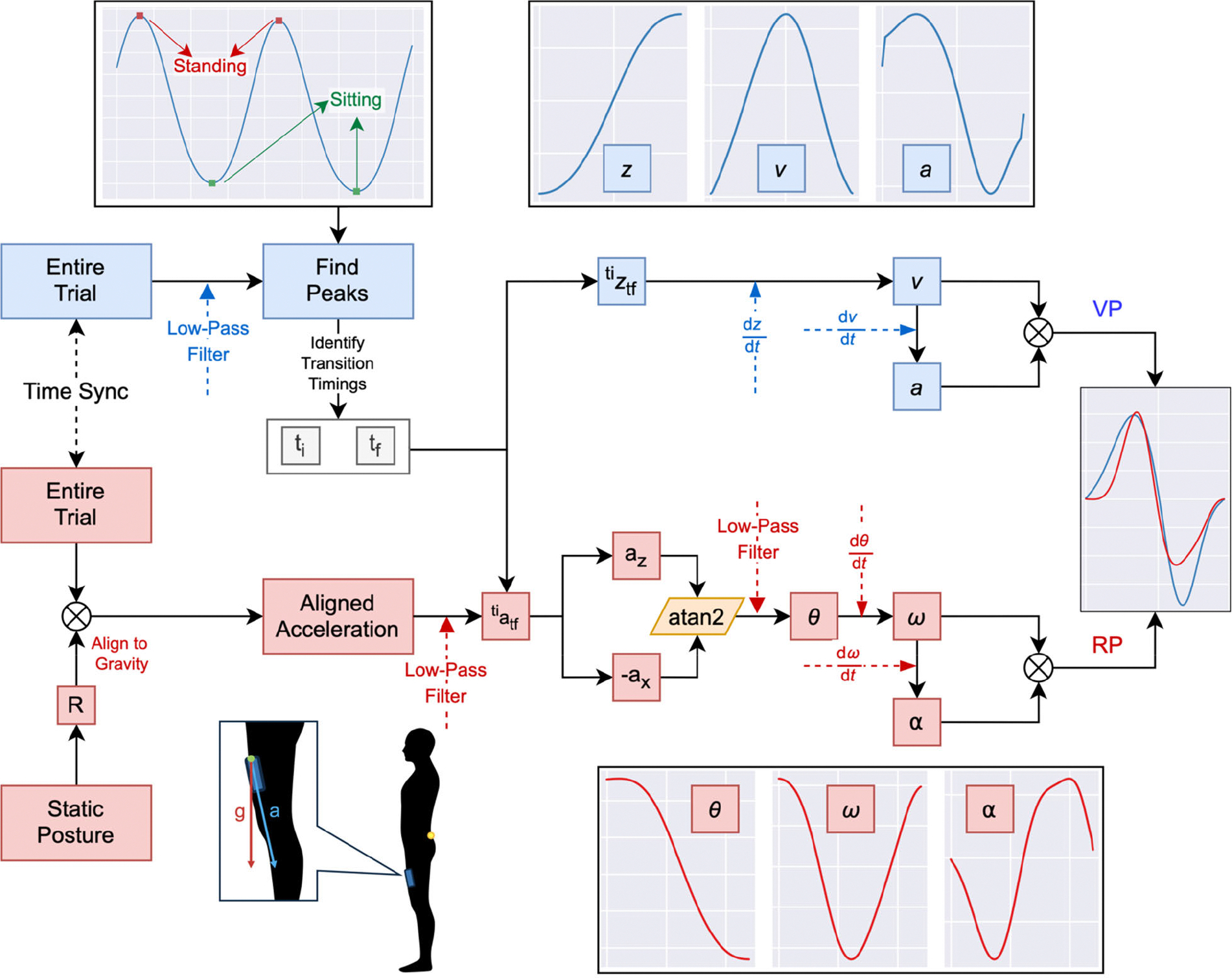
Block scheme describing the data processing employed for computing vertical (blue) and rotational (red) power. Combined processing: The two systems were time synchronized using cross-correlation. Vertical mechanical power (top, blue): the start (t_i_) and end (t_f_) of each transition was detected leveraging the local maxima and minima of the vertical displacement; once cropped, the vertical displacement of the analyzed transition (^ti^z_ff_) was numerically differentiated for obtaining vertical velocity (v) and acceleration (a). Eventually, their multiplication gave the body-mass normalized vertical mechanical power (VP). Rotational power (bottom, red): the static posture trial was leveraged for aligning the accelerometer measure by extracting the static rotation matrix (R). The aligned acceleration was trimmed from t_i_ to t_f_ (^ti^a_tf_); the angle that the thigh formed with the vertical direction (*θ*) was computed using the anterior-posterior (az) and the cranial-caudal (−ax) acceleration components as the first and second atan2 arguments, respectively. Subsequently, the angular velocity (*ω*) and acceleration (*α*) were computed via numerical differentiation, with their product being the rotational power (RP).

For the accelerometer data, accelerometer measurements were transformed to their anatomical representation by leveraging the orthostatic acquisition and a numerical implementation of the Rodrigues’ rotation formula [49]. The aligned accelerometer data were subdivided into individual transitions, leveraging the timings previously computed via motion-capture trajectories. For each transition, the cranialcaudal (*a*_*X*_*)* and anterior-posterior (*a*_*Z*_*)* axes were low-pass filtered (4^th^-order Bessel filter, cut-off = 2 Hz) and the pitch angle (*θ)* that the left thigh formed with the vertical plane throughout the transition was computed as the atan2(*a*_*Z*_, −*a*_*X*_*)*. After *θ* was further low pass filtered (4^th^-order Bessel filter, cut-off = 2.5 Hz), the corresponding angular velocity (*ω)* and acceleration (*α)* were computed via numerical differentiation. The RP was computed as the product of *α* and *ω*. The positive peak RP (RPP) was extracted from each transition. A schematic depiction of the data processing is in Fig. 2.

### D. Statistical Analysis

#### 1) Normality Assessment and Correlations:

The normality of each variable’s distribution was assessed using the Shapiro-Wilk test. Pearson’s correlation (*r)* was applied when both variables followed a normal distribution, and Spearman’s rank correlation (*α)* was used if the latter condition was not satisfied. Correlation strengths were evaluated using Evans guidelines [50]. Significance threshold was set a priori to 0.05 for all tests.

#### 2) Analytical Validation:

Analytical Validation was conducted by assessing the correlation between the individual *RPPs*, with the reference values *VPPs* computed from the motion-capture marker trajectories. The agreement between standardized *RPPs* and *VPPs* was also assessed using intraclass correlation coefficient (ICC) on individual transitions (ICC(2,1)) and at trial level (ICC(2,k)), following the convention suggested in [51].

#### 3) Clinical Validation:

To establish the relationship between RP and constructs of balance confidence and fatigue in PwMS, the 95^th^ centile of RPPs across the 30CST (*RP95c*) was computed to capture a participant’s capacity for power generation while protecting against outliers [52], [53]. The same was done for peak vertical power (*VP95c*) to assess possible discordance between the two measurement approaches. Associations between each of these 95^th^ centile measures and the number of repetitions (#Reps), MFIS total score (and the corresponding sub-scores), and ABC score were assessed using correlation.

#### 4) Acute Fatigue Analysis:

The impact of acute fatigue induced by the 30CST on RP was assessed by dividing each trial into three 10-second time periods. This time-step was chosen to highlight the possible presence of a time-varying trend in RP throughout the task, while retaining a reasonable number of transitions for each time segment. The 95^th^ centile of peak RP was computed considering transitions completed within each period. Linear regression was performed to identify significant changes in peak RP over time. Differences between time periods were assessed using a two-sided paired t-test. A significantly negative change in RP over time (confirmed via regression slope and/or difference between time periods) confirms the ability of RP to capture 30CST-induced acute fatigue. Associations between *RP95c* in each period and the ABC and MFIS scores were assessed using the appropriate correlation analyses. Findings help to highlight which kind of transition and in what portion of the task best capture the constructs of balance confidence and fatigue.

## III. Results

### A. Analytical Validation

**Fig. 3.**
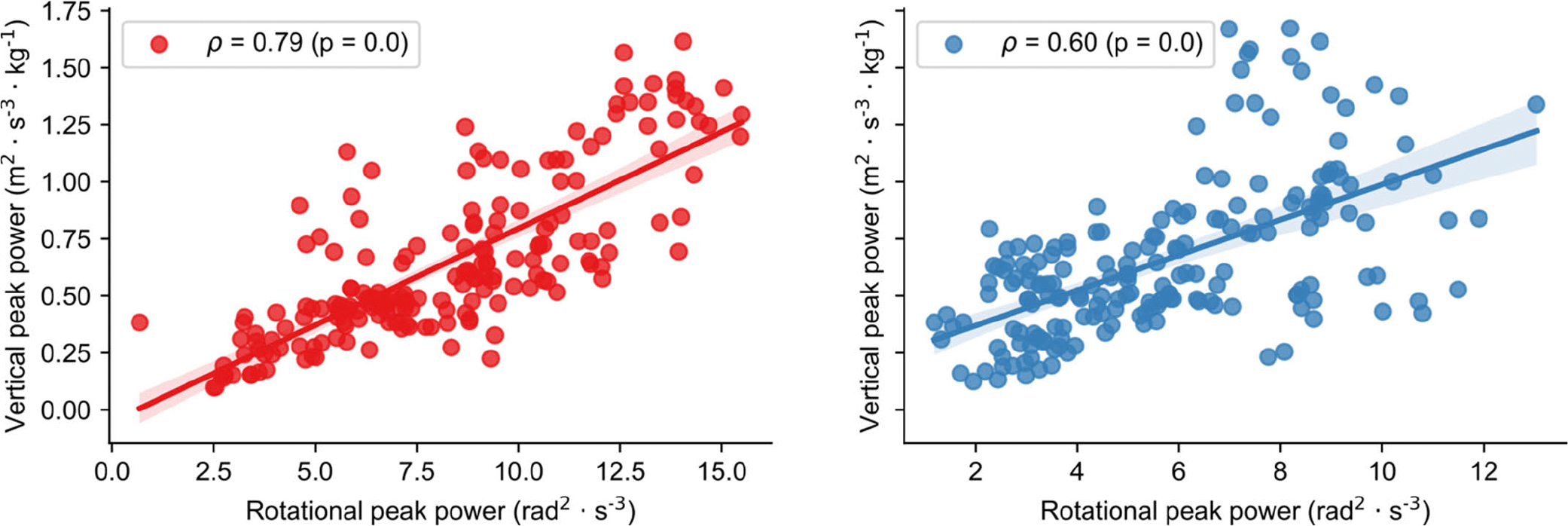
Results of the analytical validation for SI-ST (left panel) and ST-SI (right panel). Spearman’s rank correlation between the individual RPPs and VPPs showed a strong significant positive association for SI-ST (*ρ* = 0.79; p < 0.001) and a strong significant positive association ST-SI (*ρ* = 0.60, p < 0.001).

**Fig. 4.**
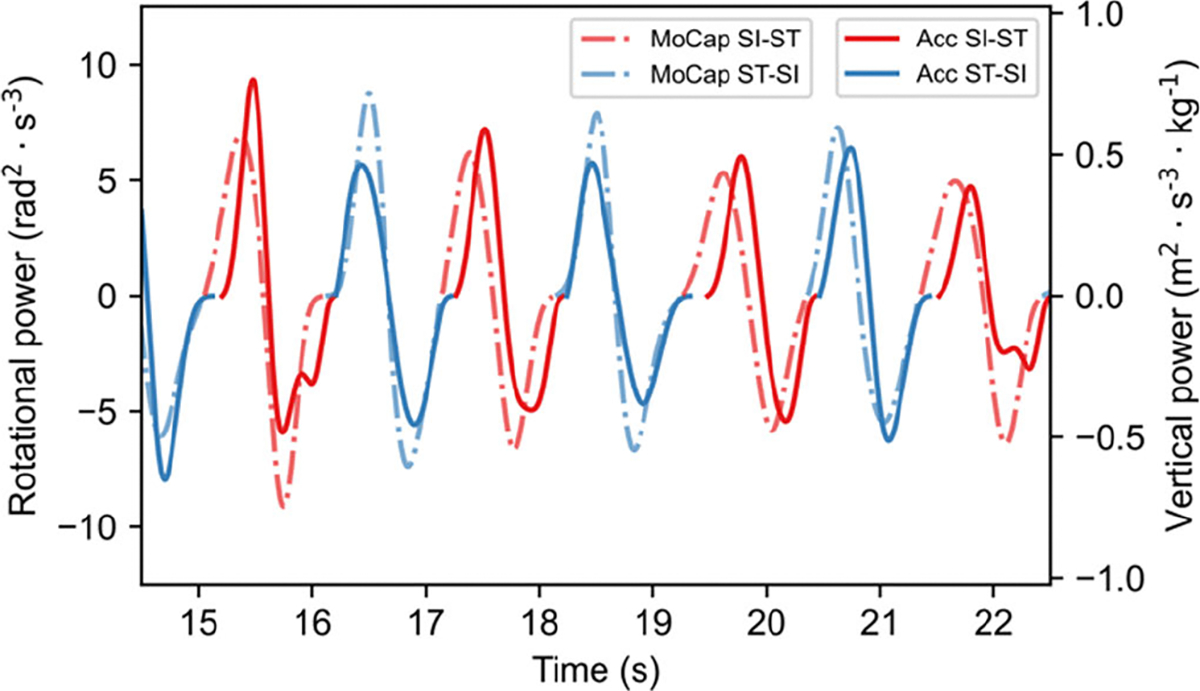
Time evolution of the individual SI-ST and ST-SI rotational power (continuous line) and vertical power (dot-dashed lines) for a subject representative of the population. There is a clear decrease in rotational peak power magnitude for SI-ST (red), whereas no significant change occurs in ST-SI (blue).

A total of 384 transitions were analyzed (# SI-ST = #ST-SI = 192), with each subject performing an average (± standard deviation) of 11.3 ± 3.6 repetitions across the 30CST. Spearman’s correlation between the individual *RPPs* and *VPPs* showed a strong significant positive association for SI-ST (*ρ* = 0.79; p < 0.001) and a strong significant positive association ST-SI (*ρ* = 0.60, p < 0.001), as depicted in Fig. 3. Individual standardized *RPPs* and *VPPs* showed a good (ICC(2,1) = 0.84; 95% CI: [0.62, 0.94]) and moderate (ICC(2,1) = 0.68; 95% CI: [0.3, 0.87]) agreement for SI-ST and ST-SI, respectively. When evaluated at trial level, the same two measures exhibited an excellent agreement (ICC(2,k) = 0.91; 95% CI: [0.76, 0.97]) for SI-ST, and a good agreement for ST-SI (ICC(2,k) = 0.81; 95% CI: [0.46, 0.93]). An example of the time series for both systems is presented in Fig. 4.

### B. Clinical Validation

After aggregation, SI-ST *RP95c* showed a strong positive correlation with #Reps (r = 0.79; p < 0.001), a strong positive correlation with the ABC total score (*ρ* = 0.68; p = 0.003), and a strong inverse correlation with MFIS (r = −0.66; p = 0.004). ST-SI *RP95c* showed a similar pattern when correlated with #Reps (very strong, r = 0.83; p < 0.001), ABC total score (moderate, *ρ* = 0.59; p = 0.013), and MFIS (moderate, r = −0.52; p = 0.034). Remarkably, #Reps showed a weaker correlation strength with ABC (*ρ* =0.61; p = 0.009) and with MFIS (r = −0.49; p = 0.047) than SI-ST *RP95c*, implying that these power-based measures may better capture mechanisms underlying balance- and fatigue-related impairment. A subsequent regression analysis was performed by including age and gender as confounders when significantly correlated with MFIS and ABC scores. The analysis showed that SI-ST *RP95c* was significantly associated for ABC (R^2^ = 0.93) and MFIS (R^2^ = 0.87), controlling for age and gender where appropriate. This was partially true for ST-SI *RP95c*, which was significantly associated with ABC (R^2^ = 0.91), but not MFIS although with high R^2^ (0.85). Further analysis of the MFIS sub-scores highlighted that SI-ST *RP95c* had an inverse relationship with the physical (moderate, r = −0.54, p = 0.016), cognitive (strong, r = −0.61, p = 0.008), and psychosocial (strong, r = −0.71, p = 0.001) domains. ST-SI *RP95c* expressed a similar trend for cognitive (moderate, r = −0.53, p = 0.029) and psychosocial (moderate, r = −0.57, p = 0.001) sub-scores. Surprisingly, ST-SI *RP95c* was not correlated with the physical sub-score of the MFIS (r = −0.39, p > 0.05). All comparisons, accounting also for the maximal-effort vertical power, are detailed in Table I.

### C. Acute Fatigue Analysis

**Fig. 5.**
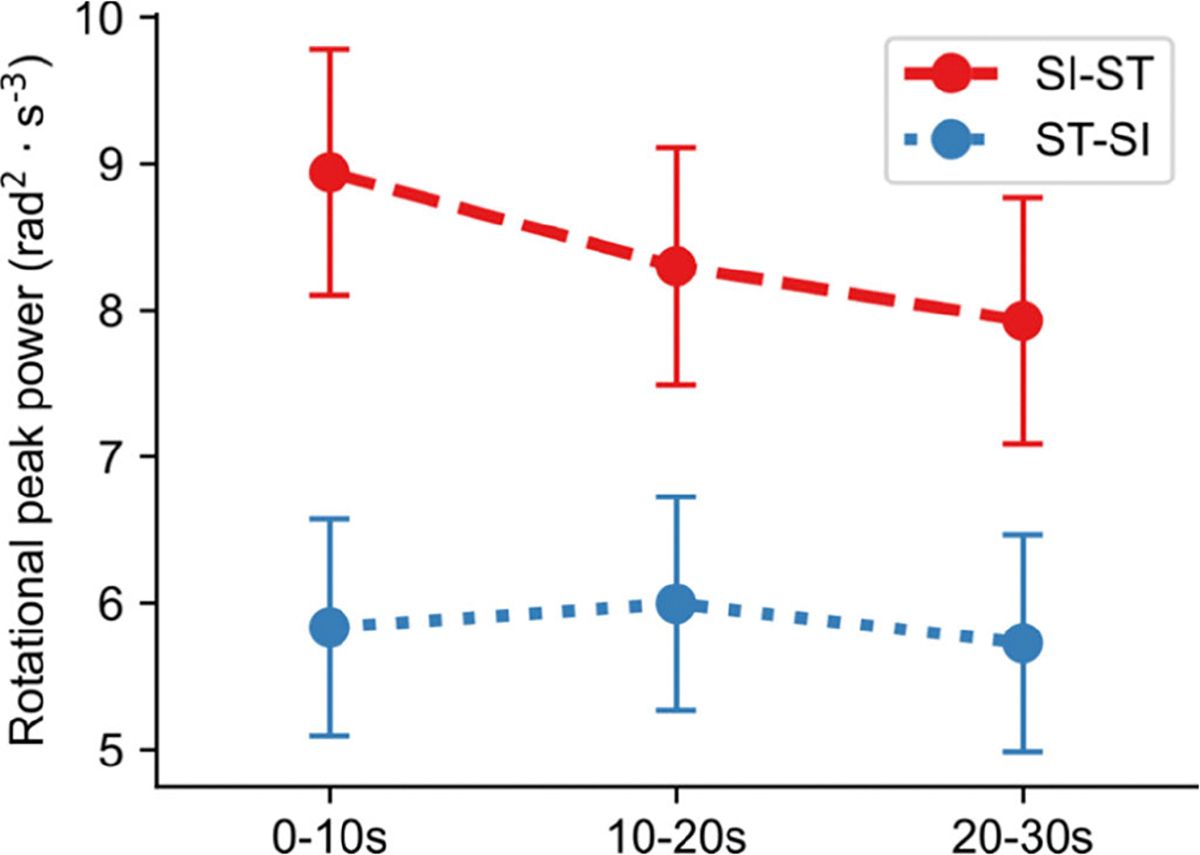
Mean RP95c (± standard error) for each 10 second period. The mean values and the corresponding standard errors are computed combining all the subjects for that given time-period.

Subsequent fatigue analysis (Fig. 5) performed by analyzing the evolution of peak power generation across the three 10-second periods, revealed a decreasing trend (slope = −0.51; p = 0.049) of the SI-ST *RP95c* across the task, with significantly higher mean values during the 0–10s interval than during the 20–30s one (t(16) = 2.40; p = 0.029). Conversely, no significant trend was observed in ST-SI power across the task (slope = −0.05; p = 0.372).

**Fig. 6.**
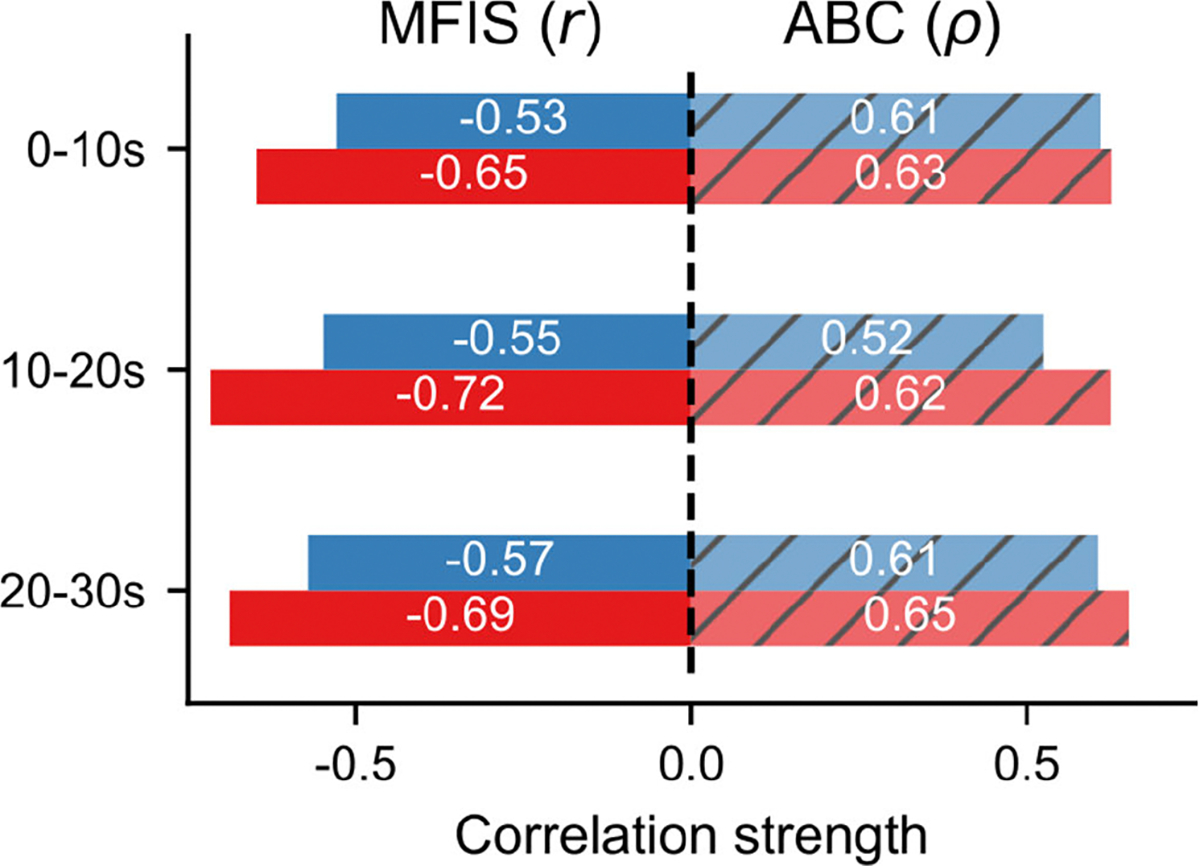
Correlation analysis performed considering only a limited portion of the task between RP95c and MFIS (left) and ABC (right) scores. The red bars refer to SI-ST, whereas the blue bars refer to ST-SI. The symbols r and *ρ* refer to Pearson’s and Spearman’s correlation coefficients, respectively.

Correlation analysis for each period (Fig. 6) highlights that *RP95c* was steadily strongly correlated with the MFIS (mean r = −0.69), with the SI-ST more capable of capturing markers of power generation underlying fatigue-related impairments than ST-SI, with a peak in correlation found at the 10–20s interval (r = −0.72, p < 0.001). The same analysis performed on ABC scores highlighted that *RP95c* was also strongly associated with balance confidence in all the three considered periods (mean *ρ* = 0.63). Correlations observed between ST-SI *RP95c* and both MFIS and ABC scores were weaker than for SI-ST in all periods (mean r = −0.55; mean *ρ* = 0.58). Interestingly, a consistent decrease in correlation between ABC scores and ST-SI *RP95c* occurs during the 10–20s interval.

## IV. Discussion

In the present study, we introduce RP as a way to quantify postural transition performance using data from a single accelerometer worn on the thigh during a 30CST. We further analytically validate this new metric relative to gold-standard optical motion capture and clinically validate its ability to capture functional impairment due to balance confidence and fatigue and acute fatigue induced by the 30CST in PwMS according to the V3 framework [54]. Validation is performedin a sample of PwMS who are ambulatory without aid and exhibit relatively low levels of impairment due to fatigue or balance confidence.

Analytical validation demonstrated a strong association and a moderate to excellent agreement between RP and gold-standard VP for both SI-ST and ST-SI transitions (Fig. 3). This finding supports the potential use of RP as a substitute for VP. This is important, given that reduced VP during postural transitions has already been associated with frailty, poor quality of life, and increased risk of falling, hospitalization, and mortality [28], [29], [30], [31], but it is difficult to assess outside of specialized laboratory environments. In contrast, RP can be assessed remotely using a single accelerometer, which could facilitate its application for more frequent assessments not only in clinical settings, but also potentially in home environments. This could enable more frequent remote monitoring of MS symptoms over time and more timely intervention. Beyond just MS, the development and validation of an algorithm for quantifying mechanical power from thigh accelerometer data could also be deployed in retrospective analyses to better understand functional impairment in large studies that have included thigh-worn, accelerometer-based activity monitors (e.g., [40], [41], [42]).

Clinical validation of the capacity to generate RP during the 30CST revealed important relationships with functional impairment due to balance confidence and fatigue (Table). Specifically, a reduced ability to generate RP was associated with decreased balance confidence and increased fatigue impact. These findings may imply a mechanistic relationship such that reduced capacity to generate RP reduces an individual’s ability to complete daily activities. Thus, measurements of RP capacity (e.g., *RP95c* during the 30CST) could potentially serve as objective markers of these impairments, and particularly because they are more strongly associated with these constructs than the traditional 30CST outcome #Reps.

While most associations were strongest for SI-ST transitions, *RP95c* and *VP95c* produced in ST-SI transitions were more strongly associated with #Reps than their counterparts in SI-ST transitions (Table I). This could indicate that the 30CST may be biased slightly toward capturing the eccentric component of power in PwMS. Such a finding may point toward an association of eccentric power with the functional endurance and strength of participants. Indeed, a deficit in eccentric control has been shown to strongly limit the functional performance of an individual, and this appears to be particularly evident for PwMS [55].

Analysis of the MFIS sub-scores revealed a moderate to high association with SI-ST *RP95c* across all domains of fatigue impact. Interestingly, this trend was confirmed only for cognitive and psychosocial scores when considering ST-SI *RP95c*. Notably, the psychosocial impact of fatigue showed the strongest relationship with *RP95c* for both transitions. While this may be due in part to the fact that this sub-score is computed from only 2 of the 21 items from the MFIS, those items are also related to the impact that fatigue has on participating in daily activities. This relationship may also be impacted by a person’s ability to generate the power required to complete those daily activities.

The analysis of acute fatigue (Fig. 5) demonstrates that RP captures the muscular fatigue induced during the 30CST [20], [32]. Specifically, acute fatigue was observed as a decay in concentric (SI-ST) *RP95c*, which is in line with previous studies leveraging electromyography [32]. Interestingly, no decay was observed for its eccentric (ST-SI) counterpart. This discrepancy may be attributed to the intrinsic nature of the two transitions involved in the maximal-effort task. While the SI-ST transition is entirely active, the ST-SI transition is partially mediated by gravity [53]. Given that RP is computed as the product of angular velocity and angular acceleration, maintaining a constant movement kinematic is more feasible when gravitational assistance is present.

Acute fatigue during the 30CST causes changes in RP generated by participants, and these changes impact the relationship between RP and functional impairment due to balance confidence and fatigue. Splitting the 30CST into three 10-second windows, associations between MFIS and SI-ST *RP95c* peak during the middle time window (Fig. 6). The fact that these enhanced associations emerge later in the task may suggest that the 30CST could provide better assessment of impairment due to fatigue than shorter tasks (e.g., five times sit-to-stand test).

Despite including analytical and clinical validation of a novel measure of mechanical power, this study is not without several limitations. First, the study relied on a secondary analysis of a sample composed of a small number of PwMS, with no measure of the disease stage other than self-reported PDDS. In turn the sample presented limited mobility impairment (moderate-to-high balance confidence, low impairment due to fatigue, and low patient-determined disease stage). This should be addressed in future studies in samples with more severe mobility impairment. Moreover, in the current study spasticity was not assessed. Given the relationship between its severity and patient impairment, future studies on RP in PwMS should also investigate its impact. Furthermore, the study design prevents the assessment of test-retest reliability, and inter- and intra-participant variability. Next, despite the sensor was reoriented to align it with gravity while standing, no assumption can be made about the effect of each individual anatomical variability. In addition, we computed RP from one limb only, possibly hiding bilateral asymmetries. Finally, we consider only data from an in-lab 30CST, which may not reflect real-world performance and be influenced by the observer effect. Considering the association and agreement with VP that was found in the current study, future work should aim to examine 30CSTs performed outside the laboratory environment while unsupervised. Nonetheless, while the 30CST is a functional assessment with relatively low barriers to deploying outside of clinical contexts, doing so is still more of a burden for participants than purely passive monitoring of daily activities (e.g., [56]). Future work should consider quantifying RP during free-living postural transitions and exploring how it relates to RP computed during the 30 CST. Given the promising results presented here, future work could also consider embedding RP as a part of a comprehensive daily mobility index that can be used to describe lower limb motor capacity across activities and how they contribute to general mobility impairment.

## V. Conclusion

In this study, we introduce rotational power as new method for characterizing postural transition performance during the 30CST from a single thigh-worn accelerometer. To ensure that the method is fit for purpose, we present results from analytical and clinical validation in a sample of PwMS. Rotational power is shown to agree well with gold-standard vertical power derived from optical motion capture and be strongly associated with functional impairment due to balance confidence and fatigue. Finally, we demonstrate that rotational power during the SI-ST phase of the transition is able to capture acute fatigue induced by the 30CST, further emphasizing its utility as a marker of postural transition performance. These findings point toward future use of rotational power as a marker of functional impairment in PwMS, and potentially in other populations with symptoms impacting balance and mobility.

## Figures and Tables

**TABLE I:** Correlation Analysis for the Maximal-Effort Rotational Power (RP95c) and Reference Vertical Power (VP95c) With Clinical Measures

	*RP95c* (SI-ST / ST-SI)	*VP95c* (SI-ST / ST-SI)
**#Reps (*r*)**	0.79* / 0.83*	0.70* / 0.78*
**ABC (*ρ*)**	0.68* / 0.59*	0.73* / 0.51*
**MFIS (*r*)**	−0.66* / −0.52*	−0.61* / −0.62*

Asterisk: p < 0.05; #Reps: number of repetitions during the 30CST; ABC: activity-specific balance confidence scale; MFIS: modified fatigue impact scale.
